# Identification and functional analysis of five hub lncRNAs associated with oncogenesis of Hepatocellular Carcinoma

**DOI:** 10.1371/journal.pone.0321875

**Published:** 2025-04-30

**Authors:** Zhaomin Deng, Lu Pu, Yongxin Ma, Hao Jiang

**Affiliations:** 1 Laboratory for Aging and Cancer Research, National Clinical Research Center for Geriatrics, West China Hospital, Sichuan University, Chengdu, China; 2 Laboratory for Aging and Cancer Research, Frontiers Science Center for Disease-related Molecular Network, West China Hospital, Sichuan University, Chengdu, China; 3 Department of Medical Genetics, State Key Laboratory of Biotherapy, West China Hospital, Sichuan University, Chengdu, China; 4 Department of Medical Genetics, Frontiers Science Center for Disease-related Molecular Network, West China Hospital, Sichuan University, Chengdu, China; 5 Department of Gastroenterology and Hepatology, West China Hospital, Sichuan University, Chengdu, China; 6 Sichuan University-University of Oxford Huaxi Joint Centre for Gastrointestinal Cancer, Frontiers Science Center for Disease-Related Molecular Network, West China Hospital, Sichuan University, Chengdu, China; 7 State Key Laboratory of Respiratory Health and Multimorbidity, West China Hospital, Sichuan University, Chengdu, China; Children's Cancer Institute Australia, AUSTRALIA

## Abstract

Liver cancer stands as one of the most pervasive and aggressive malignant neoplasms on a global scale, with hepatocellular carcinoma (HCC) accounting for over 90% of primary liver cancer cases. It has been documented that long non-coding RNA (lncRNA) plays a significant role in various pathological contexts and serves as a pivotal determinant in numerous malignancies, including HCC. Leveraging mRNA sequencing data and lncRNA expression profiles from 346 HCC samples and 50 pairs of adjacent normal samples sourced from the TCGA database, a comprehensive bioinformatics analysis was conducted. The dataset was randomly partitioned into a training subset and a validation subset. This rigorous analysis aimed to pinpoint the lncRNAs intricately linked to the oncogenesis of HCC, unveiling distinct expression patterns of these lncRNAs between normal liver and HCC samples across different stages. Moreover, the construction of a lncRNA-mRNA co-expression network led to the identification of five central lncRNA hubs (AC091057, AC099850, AC012073, DDX11-AS1, and AL035461). Subsequently, these five lncRNAs were validated using the independent validation set. In summary, this investigation successfully discerned five lncRNAs closely associated with the oncogenesis of HCC, thereby shedding light on their potential utility as diagnostic or therapeutic targets for this formidable disease.

## Introduction

Hepatocellular carcinoma (HCC), the predominant form of liver cancer, is a prevalent and life-threatening condition responsible for more than 70% of all liver cancer cases [1–3]. Its prevalence and mortality rates are increasing globally, particularly in the United States and Europe [4,5]. HCC predominantly affects men and is associated with various factors, including chronic inflammatory diseases, nitrites, aflatoxins, genetic disorders, alcohol consumption, and tobacco smoking [6–10]. Despite significant advancements in HCC diagnosis, such as staging systems and characteristic imaging studies [11–15], the prognosis and five-year survival rate remain disappointingly low [7,16]. Therefore, the identification of new biomarkers for HCC diagnosis, therapy, and prognosis is of utmost urgency.

The development and progression of HCC are driven by complex epigenetic mechanisms that alter gene expression patterns. Among these mechanisms, long non-coding RNAs (lncRNAs) and DNA methylation have emerged as critical regulators. LncRNAs, a class of non-coding RNAs exceeding 200 nucleotides in length [17–19], have garnered considerable attention due to their regulatory roles in carcinogenesis and their intricate links to gene expression [20–22]. Similarly, DNA methylation, a key epigenetic modification, plays a pivotal role in HCC progression and treatment by exerting transcriptional control over gene expression and chromatin conformation [23]. Both lncRNAs and DNA methylation can independently influence HCC development, and accumulating evidence suggests that these epigenetic mechanisms may also interact to modulate HCC pathogenesis.

Emerging evidence suggests that lncRNAs are intricately linked to gene expression and hold promise as potential biomarkers for HCC [22,24–27]. Moreover, several lncRNAs, such as GHET1, DBH-AS1, and HOST2, have been implicated as tumor suppressors in HCC through diverse molecular pathways [28–31]. Similarly, DNA methylation, a key epigenetic modification, plays a pivotal role in HCC progression and treatment [32,33] by exerting transcriptional control over gene expression and chromatin conformation [34,35]. Aberrant DNA methylation, for example, has been shown to drive HCC pathogenesis by repressing tumor suppressor genes [35–37] and holds promise as a diagnostic tool [38], making DNA methylation patterns informative biomarkers for HCC [33]. Both lncRNAs and DNA methylation can independently influence HCC development, and accumulating evidence suggests that these epigenetic mechanisms may also interact. For example, certain lncRNAs can regulate DNA methylation patterns by recruiting DNA methyltransferases or modulating chromatin remodeling complexes, thus influencing gene expression and tumor progression [39]. This interplay between non-coding RNAs and DNA methylation highlights the complexity of epigenetic regulation in HCC pathogenesis and offers novel avenues for potential therapeutic strategies [40].

Given the growing recognition of lncRNAs as key players in tumor development and their potential clinical applications, and recognizing that their function may be intertwined with other epigenetic regulators, this study aims to comprehensively analyze lncRNAs associated with the diagnosis and prognosis of HCC patients. By identifying and characterizing these lncRNAs, this research seeks to contribute to the early detection and treatment of HCC, offering improved outcomes for affected individuals.

## Results

### LncRNAs in normal liver and different stages HCC samples

**Fig 1:**
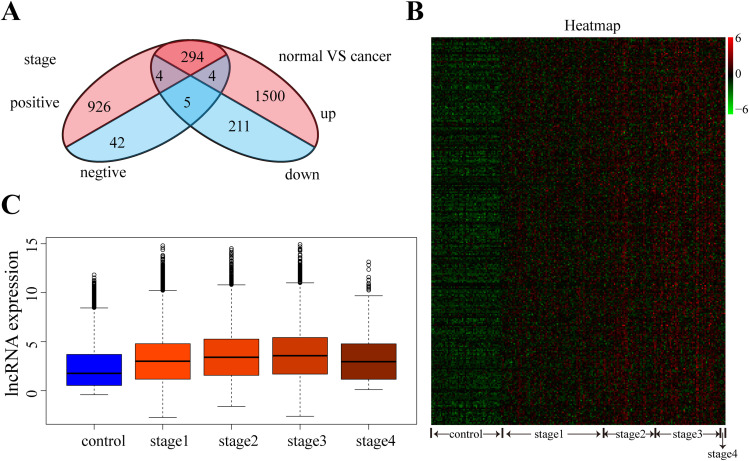
Identification and Expression Patterns of lncRNAs Associated with HCC Tumor Progression. (A) displays the overlap between differentially expressed lncRNAs in hepatocellular carcinoma (HCC) and lncRNAs correlated with HCC stages. The Venn diagram illustrates the intersection between lncRNAs that are differentially expressed in HCC samples compared to normal liver tissues and those that exhibit expression patterns correlated with different stages of HCC progression. In Panel (B), the heatmap depicts the correlation of lncRNAs with normal liver and HCC samples across four stages of HCC progression. Each row represents a differentially expressed lncRNA, while each column represents a sample categorized into one of four stages of HCC. The color gradient indicates the expression levels of lncRNAs, with warmer colors representing higher expression levels and cooler colors indicating lower expression levels. Panel (C) Quantification of expression differences of 294 IncRNAs in normal liver and HCC samples across the four stages of HCC progression. The bar plot illustrates the mean expression levels of lncRNAs in normal liver tissues compared to HCC samples at each stage. The y-axis represents the average expression levels, while the x-axis denotes the four stages of HCC progression.

To identify differentially expressed lncRNAs in HCCsamples compared to their normal paracancerous counterparts, we employed the EdgeR algorithm, resulting in the identification of 1798 upregulated lncRNAs and 220 downregulated lncRNAs in HCC samples relative to their normal adjacent tissues (FDR < 0.05) (Fig 1A). Additionally, we observed 1224 positively correlated lncRNAs and 51 negatively correlated lncRNAs with HCC stage (p-value < 0.05) (Fig 1A). Subsequently, we identified 294 highly expressed lncRNAs in HCC relative to adjacent tissues that were positively correlated with HCC stage (Fig 1A). Due to the limited number of lncRNAs in the remaining three sets, further analysis was not pursued. As depicted in Fig 1B and C, these 294 lncRNAs exhibited distinct expression patterns in normal liver and HCC samples across various stages. Notably, the expression of these lncRNAs escalated with HCC tumor progression, although this trend was less discernible in the limited stage 4 samples.

### LncRNA-mRNA co-expression network

**Fig 2:**
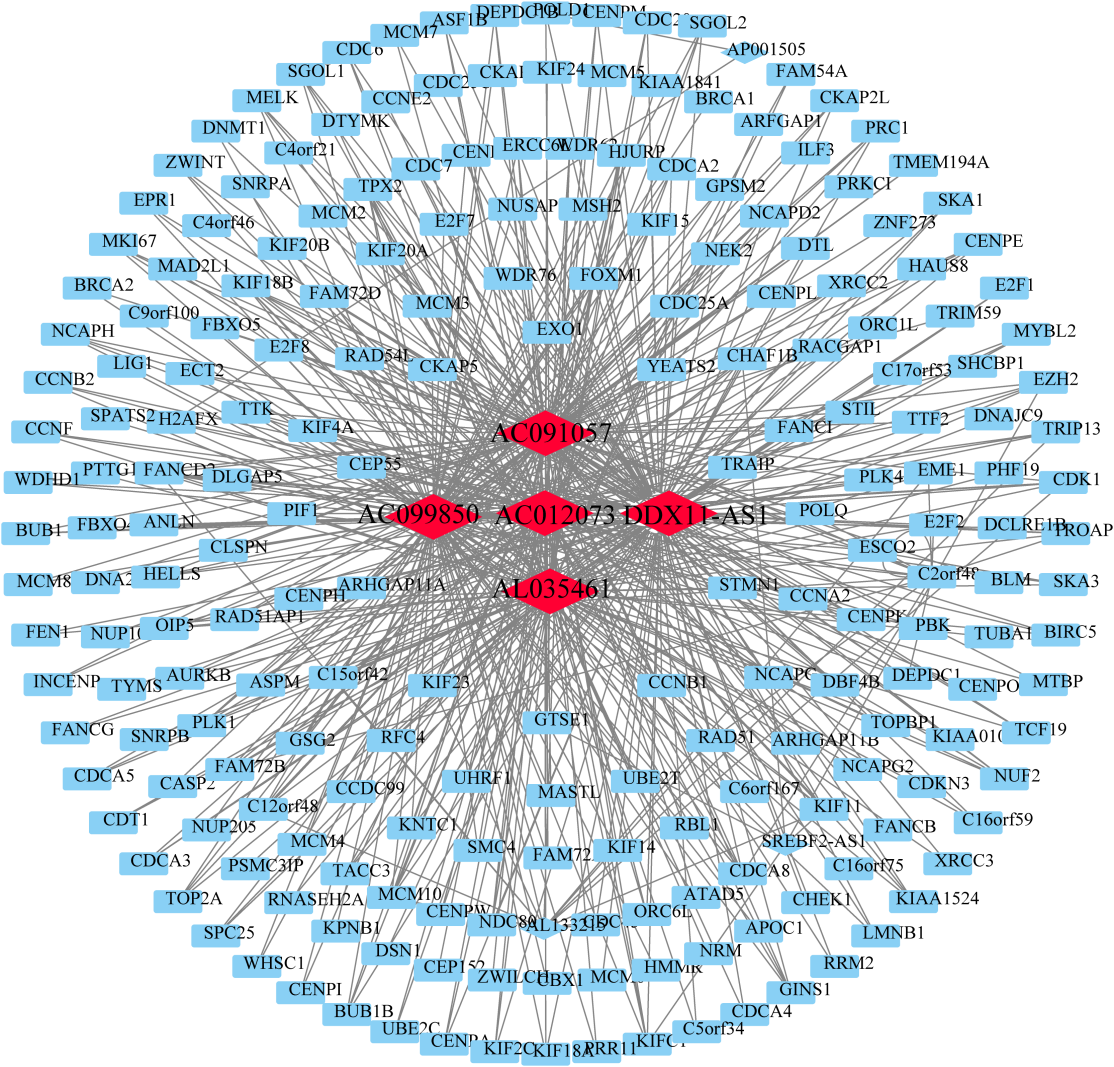
Co-expression network of lncRNAs and mRNAs. The co-expression network visually depicts the intricate interplay between lncRNAs and mRNAs in the context of hepatocellular carcinoma (HCC). Each node in the network represents either a lncRNA (depicted in red) or an mRNA (depicted in blue), and the edges between nodes illustrate significant Spearman correlations identified through rigorous statistical analysis. The size of each node corresponds to the degree of connectivity within the network, reflecting the number of correlated partners for each lncRNA or mRNA.

In order to elucidate the pivotal regulatory functions of lncRNA and mRNA in the biological processes of HCC, we employed Spearman correlation analysis to identify mRNAs that were significantly associated with the previously screened lncRNAs (Spearman Correlation, P < 0.05). Subsequently, a co-expression network of lncRNA-mRNA was constructed based on the correlations obtained. Within this network, we identified a prominent subnetwork (Fig 2) comprising five hubs of lncRNAs (AC091057, AC099850, AC012073, DDX11-AS1, and AL035461). Remarkably, the lncRNAs in these hubs exhibited similar expression patterns to a substantial number of mRNAs, and their expression patterns were highly correlated with each other. Notably, previous studies have highlighted the significant role of DDX11-AS1 in HCC tumorigenesis, suggesting its potential as a therapeutic target for HCC [41]. This finding implies that the remaining four lncRNAs may also play similarly crucial roles in the context of HCC.

### GO pathway analyses in all the mRNAs associated with five hub lncRNA expression

**Fig 3:**
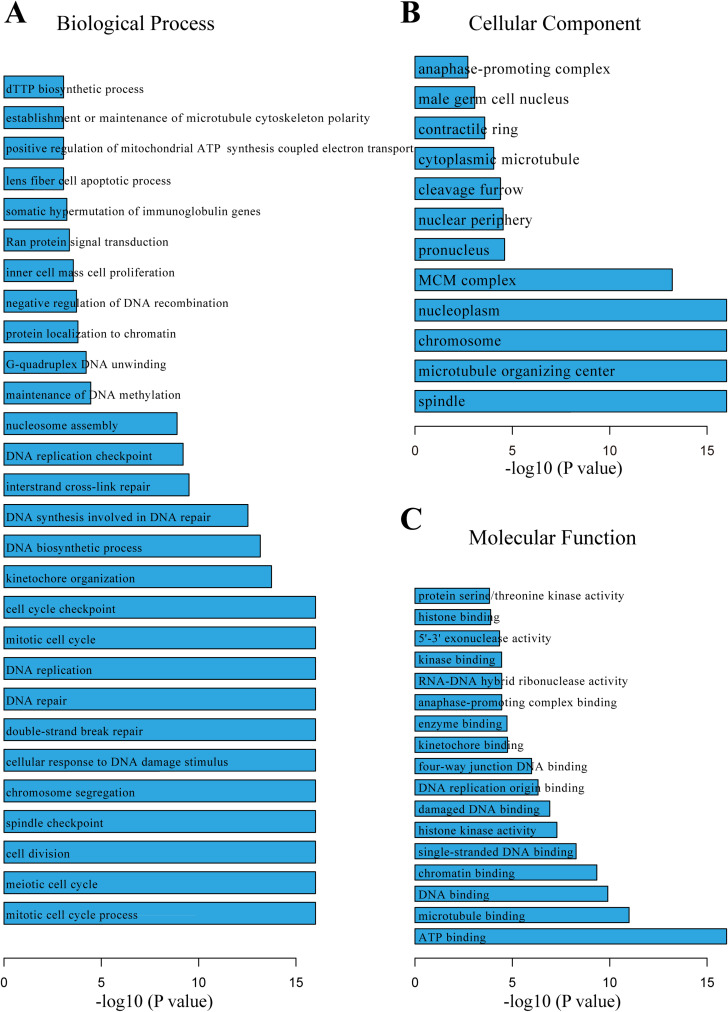
Gene Ontology (GO) enrichment analysis of target genes associated with five hub lncRNAs. The bar charts illustrate the results of GO enrichment analysis conducted to elucidate the functional implications of the target genes associated with five identified hub lncRNAs in the context of hepatocellular carcinoma (HCC). Panel A represents the enriched biological processes, panel B depicts the enriched cellular components, and panel C shows the enriched molecular functions. Each bar in the charts corresponds to a specific GO term, and the height of the bars reflects the significance or enrichment score of the corresponding GO term. The color intensity of the bars indicates the level of significance, with darker shades representing higher significance levels.

To further explore the role of the aforementioned five lncRNAs in HCC development, we utilized GO functional enrichment analysis to examine the mRNAs exhibiting similar expression patterns to these five lncRNA hubs. The genes were classified into three distinct groups based on the Gene Ontology (GO) analysis: biological processes (BP) (Fig 3A), cellular components (CC) (Fig 3B), and molecular functions (MF) (Fig 3C). Within the biological process (BP) category, these mRNAs demonstrated significant enrichment in cell cycle-related pathways, such as cell cycle, cell division, and DNA replication. Additionally, it is noteworthy that these mRNAs also exhibited enrichment in pathways related to the maintenance of DNA methylation and somatic hypermutation of immunoglobulin genes. This observation suggests potential involvement of these five lncRNAs in DNA methylation and somatic mutation processes in HCC patients.

### Validation the five lncRNAs in independent data

**Fig 4:**
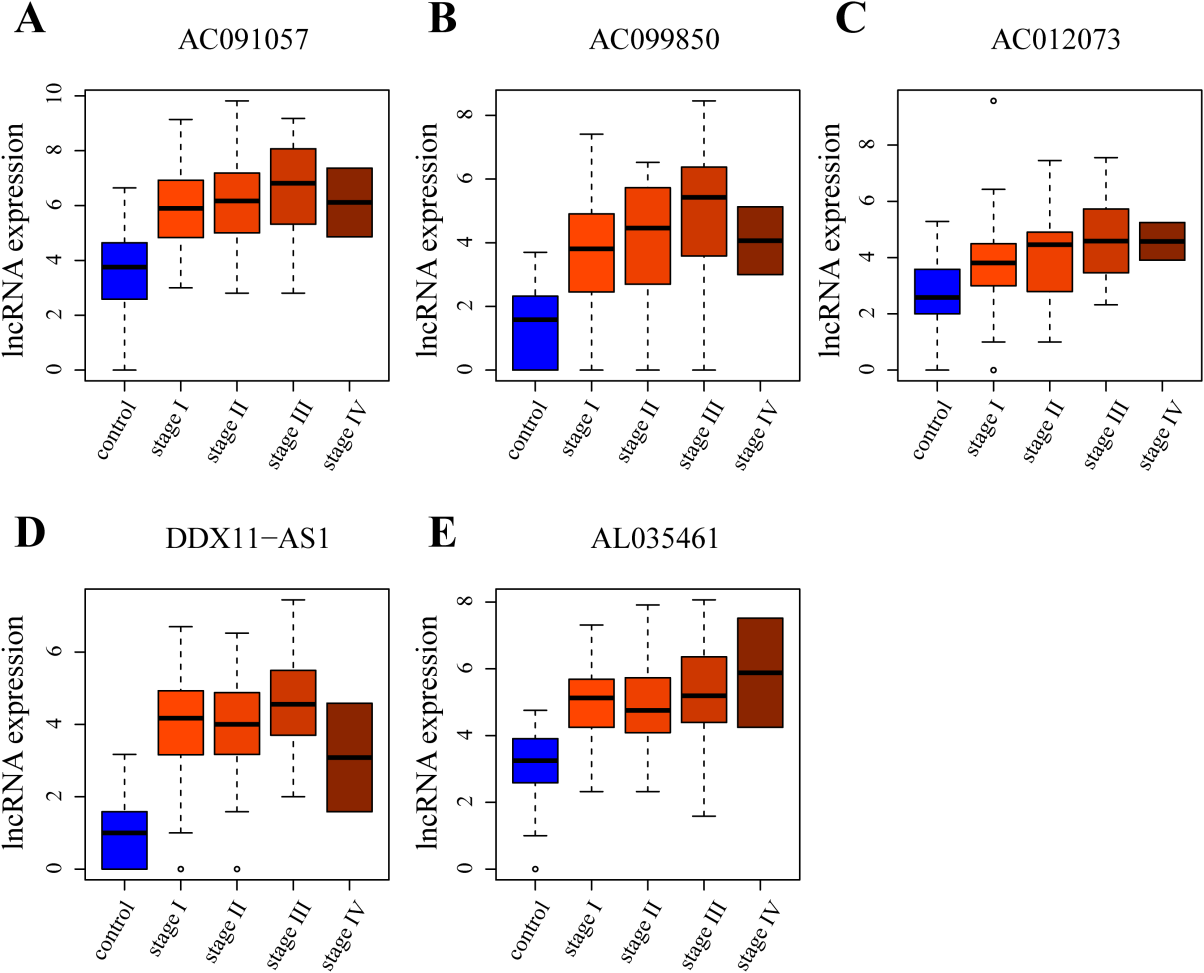
Expression levels of five hub lncRNAs in validation data. The bar plots display the expression levels of the five identified hub lncRNAs, namely (A) AC091057, (B) AC099850, (C) AC012073, (D) DDX11-AS1, and (E) AL035461, in an independent validation dataset related to hepatocellular carcinoma (HCC). Each bar represents the expression level of the corresponding lncRNA, with the y-axis indicating the expression values.

The expression profiles of these five lncRNAs were confirmed in the validation dataset. Remarkably, the lncRNAs AC091057, AC099850, AC012073, DDX11-AS1, and AL035461 exhibited a consistent upregulated expression pattern in HCC compared to normal tissue, as illustrated in Fig 4A-4E. Notably, the expression levels of AC091057, AC099850, AC012073, and AL035461 were all positively correlated with the stage of HCC. While this trend was less evident for DDX11-AS1, it is noteworthy that its expression was notably lower in early-stage HCC compared to advanced-stage HCC. Thus, these five lncRNAs consistently maintained their expression patterns in the validation dataset, consistent with the findings from the original test dataset.

### Survival analysis for the five lncRNAs

**Fig 5:**
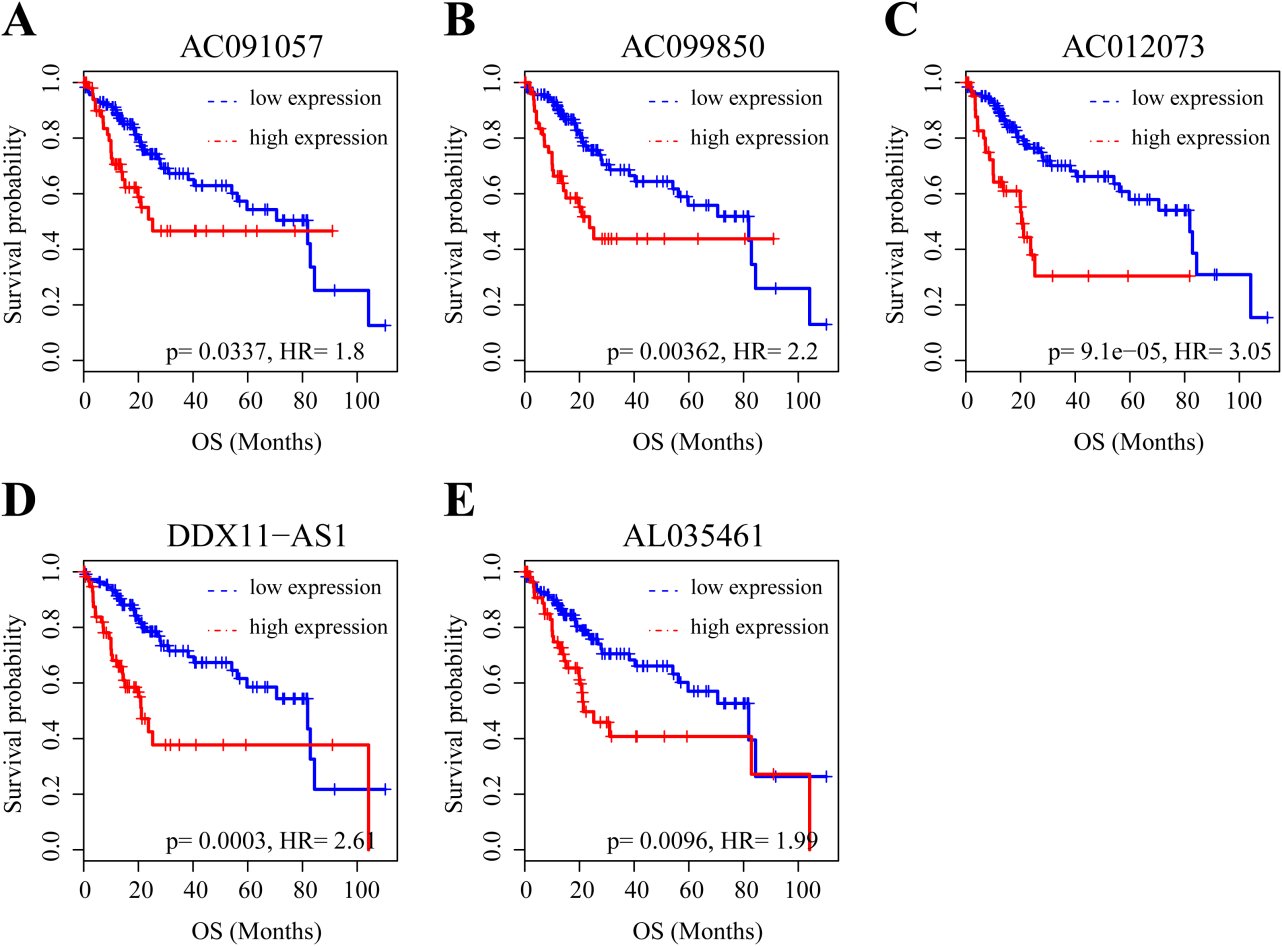
Kaplan-Meier curves demonstrating the relationship between the five lncRNAs and overall survival rate. The Kaplan–Meier curves illustrate the association between the expression levels of the five identified lncRNAs, namely (A) AC091057, (B) AC099850, (C) AC012073, (D) DDX11-AS1, and (E) AL035461, and the overall survival rates of patients with hepatocellular carcinoma (HCC). Each curve represents the probability of overall survival over a defined time period, stratified based on the expression level of the corresponding lncRNA. The x-axis represents the survival time in months, while the y-axis indicates the probability of survival.

Finally, the association between the long non-coding RNA (lncRNA) of these five central nodes and the overall survival (OS) of HCC patients was examined. Employing Kaplan-Meier and log-rank analyses revealed that the five lncRNAs could be stratified into two cohorts with markedly disparate prognoses based on their expression profiles. Furthermore, even after adjusting for gender, disease stage, and age, the expression levels of these five lncRNAs remained significantly correlated with OS in HCC patients (see Fig 5). These findings further underscore the potential relevance of the identified five lncRNAs to the prognosis of HCC progression.

## Discussion

In this study, a total of 294 lncRNAs were identified as exhibiting high expression levels in HCC compared to adjacent tissues, and their expression was found to be positively correlated with the stage of HCC. These 294 lncRNAs displayed distinct expression patterns in normal liver and HCC samples at various stages, with their expression levels increasing concomitantly with the progression of HCC tumors. Focusing on this subset of lncRNAs, we further identified five key “hub” lncRNAs (AC091057, AC099850, AC012073, DDX11-AS1, and AL035461) based on their high expression, positive correlation with stage, and extensive co-expression with protein-coding genes. These hubs are particularly interesting as their expression patterns were strongly correlated with a substantial number of mRNAs, implying potential coordinated regulatory relationships. This co-expression suggests that these lncRNAs may act as central regulators within gene networks that contribute to HCC pathogenesis.

One of the identified hubs, DDX11-AS1, has been previously studied in the context of HCC. This lncRNA is a non-overlapping transcript originating from the DDX11 gene, which encodes a DNA-dependent ATPase and helicase crucial for DNA replication [42]. Prior research has indicated that DDX11-AS1 plays a significant role in HCC oncogenesis, potentially serving as a therapeutic target [43]. Our findings align with these previous observations, further supporting the clinical relevance of this lncRNA. While the other four lncRNA hubs (AC091057, AC099850, AC012073, and AL035461) also exhibited a positive correlation with HCC stage, albeit less pronounced for DDX11-AS1, relatively little is known about their specific functions. This highlights a significant gap in our understanding of HCC biology and underscores the need for future studies to investigate the roles of these understudied lncRNAs. For instance, little is known about the mechanisms by which these lncRNAs are regulated or how they interact with other cellular components.

To gain insights into the potential functions of these lncRNA hubs, we performed Gene Ontology (GO) enrichment analysis on the mRNAs that showed similar expression patterns. This analysis revealed a strong enrichment for genes involved in essential cellular processes, including tissue and organ morphogenesis, cell survival, proliferation, differentiation, apoptosis, and migration. Notably, cell division was a particularly prominent category [44–46]. These results suggest that these lncRNA-mRNA interactions may contribute to HCC development by influencing fundamental cellular processes that are dysregulated in cancer. Furthermore, these molecular function pertained to the biochemical activities of gene products, while cellular component referred to the subcellular locations where these gene products are active [44]. The intricate interplay between these categories underscored the multifunctional nature of proteins and their involvement in diverse molecular activities and interactions within the cell.

The expression of the five lncRNA hubs was also significantly correlated with overall survival (OS) in HCC patients, pointing to their potential as prognostic biomarkers. However, it is crucial to interpret these findings with caution, given that our survival analysis was based on a subset of the original TCGA dataset, meaning that the dataset and split had not been from the same source, so these OS findings should be interpreted with caution. Further validation in independent, external cohorts is essential to confirm the prognostic value of these lncRNAs. Given the established role of cell invasion, metastasis, and tumor recurrence in HCC-related mortality, the observed correlation with OS suggests that these five lncRNAs (AC091057, AC099850, AC012073, DDX11-AS1, and AL035461) may be involved in HCC metastasis. However, it’s important to acknowledge that our validation was performed using a subset of the original TCGA dataset and we recognize it would have been stronger if the dataset and split had not been from the same source, so these OS findings should be interpreted with caution. As an in silico analysis, we have identified potential lncRNA regulators of HCC but have not experimentally validated their function. Further studies are needed to validate these results in independent, external cohorts.

## Conclusions

In this study, a cohort of HCC patients revealed the identification of five lncRNAs exhibiting closely correlated expression profiles linked to tumor progression. Notably, among these, lncRNA DDX11-AS1 has been documented to exert a pivotal role in HCC tumorigenesis, thereby presenting a potential therapeutic target for HCC. This finding implies that the remaining four lncRNAs may share analogous functional roles. Through comprehensive enrichment analysis, it has been discerned that these five lncRNAs may be implicated in methylation and mutational events within the context of HCC. Furthermore, these lncRNAs have been associated with the prognostic outcomes of HCC patients. As such, these five lncRNAs warrant further in-depth investigation.

## Methods

### LncRNA data source

In this study, the lncRNA expression profiles of 346 HCC specimens and 50 corresponding paraneoplastic samples were employed. All these lncRNA expression profiles and associated clinical data were sourced from the TCGA database [47]. For the purposes of this research, the 346 patients were randomly allocated into training and validation groups, each comprising 173 individuals. The clinical data for both groups can be found in Table 1. It is noteworthy that to obtain the lncRNA data, the RNAseq counts were initially obtained from the TCGA database, and subsequently, the desired lncRNA expression profiles were extracted based on the lncRNA information compiled in the GENCODE database [48] (https://www.gencodegenes.org/).

**Table 1:** The clinical characteristics for training group and validation group.

Covariates	Category	Training group	Validation group	*P-value*
		(n = 173)	(n =173)	
Age*	Mean ± SD	58.19±13.48	60.30±13.04	0.14
	Median	61	61	
Gender	Male	124	49	0.13
	Female	110	63	
Vital status	Alive	59	114	0.65
	Dead	54	119	
Survival time*	Mean ± SD	784.07±727.34	807.25±709.28	0.76
	Median	555	644	
stage	I	76	84	0.45
	II	35	43	
	III	47	34	
	IV	3	2	
	NA	12	10	
grade	I	28	23	0.85
	II	79	84	
	III	60	57	
	IV	5	7	
	NA	1	2	

*T-test was used to analyze the statistical difference of clinical index between the two groups.

### Data preprocessing

In the preprocessing of lncRNA data, an initial step involved the exclusion of any lncRNA that was absent in more than half of the samples. This stringent criterion was implemented to ensure the quality and reliability of the dataset. For lncRNAs missing in less than 50% of the samples, we employed the K-nearest neighbor imputation method to estimate and fill in the missing values. By adopting this approach, we aimed to maximize the utilization of available data while mitigating the impact of missing values on the subsequent analyses.

### Identification of differential lncRNA

Differentially expressed lncRNAs between normal and tumor samples in HCC were identified using the EdgeR package [49]. To ensure robust selection, we applied stringent criteria: a false discovery rate (FDR) less than 0.05 and an absolute log2 fold change (|log2FC|) greater than 1.0. These criteria allowed us to effectively pinpoint lncRNAs that exhibited significant differential expression between the two sample groups.

Furthermore, to identify lncRNAs correlated with HCC tumor stage, we performed Spearman correlation analysis between lncRNA expression levels and tumor stage information. LncRNAs with a Spearman correlation coefficient greater than 0.3 or less than -0.3 and a p-value less than 0.05 were considered significantly correlated with tumor stage and selected for further analysis.

### Selection of lncRNA Hubs

Prior to constructing the lncRNA-mRNA co-expression network, we identified five lncRNAs as “hubs” based on the following criteria, designed to select lncRNAs with potentially central regulatory roles in HCC: (1) High expression in HCC tumors relative to normal tissue (FDR < 0.05); (2) Significant positive correlation with HCC stage (p-value < 0.05); and (3) High degree of connectivity, as measured by number of co-expressed mRNAs identified through Spearman correlation analysis. Specifically, we selected the five lncRNAs with the highest number of co-expressed mRNAs that met the first two criteria.

### Construction of LncRNA-mRNA co-expression network

To further explore the regulatory relationships between lncRNAs and mRNAs, we conducted Spearman correlation analysis to identify mRNAs that exhibited significant associations with the previously identified lncRNAs. The Spearman correlation coefficient was calculated to evaluate the strength and direction of the relationships between individual lncRNAs and mRNAs, with statistical significance set at a threshold of P < 0.05. This rigorous analysis allowed us to pinpoint mRNA transcripts that were closely correlated with specific lncRNAs, providing insights into potential regulatory interactions between these two classes of transcripts.

Subsequently, based on the significant correlations identified through Spearman correlation analysis, we constructed a comprehensive co-expression network illustrating the relationships between lncRNAs and their associated mRNA targets. The co-expression network visually represents the regulatory connections between lncRNAs and mRNAs, highlighting potential regulatory modules and functional relationships within the transcriptome. By integrating information on the strength and significance of correlations, this network analysis offers a systematic view of the complex interplay between lncRNAs and mRNAs, shedding light on potential regulatory mechanisms and functional implications underlying these interactions.

### Survival analysis

The estimation of survival curves was meticulously performed utilizing the Kaplan-Meier method, a widely recognized approach in survival analysis, to assess the probability of survival over time. Subsequently, comparisons between survival curves were conducted using the log-rank test [50], a statistical test that evaluates differences in survival distributions between groups. This method provided valuable insights into the overall survival patterns associated with different lncRNA expression profiles in the context of the disease.

Furthermore, to evaluate the independent prognostic significance of the identified lncRNAs, we employed a multivariate Cox proportional-hazards regression model. This sophisticated statistical technique allowed us to assess the impact of lncRNA expression levels on patient outcomes while adjusting for potential confounding factors such as age and gender. By incorporating these clinical variables into the analysis, we aimed to delineate the unique prognostic value of the lncRNAs beyond traditional clinical parameters, providing a more comprehensive understanding of their predictive power in the HCC setting.

### Functional enrichment analysis

In order to unravel the biological relevance and functional implications of the identified lncRNAs, we conducted functional enrichment analysis to elucidate the disrupted functional categories significantly enriched in genes associated with these lncRNAs. For this purpose, we utilized the GO-function tool [51–53], a powerful bioinformatics resource that helps identify and categorize gene functions based on Gene Ontology terms. By analyzing the enriched functional terms, we aimed to gain insights into the molecular mechanisms underlying the association between lncRNAs and disease pathology, shedding light on potential biological processes and pathways influenced by these regulatory molecules. This comprehensive analysis provided a deeper understanding of the functional roles of lncRNAs in the context of the disease, facilitating the identification of key biological processes and pathways involved in HCC progression.
